# Top-Down Prioritization of Salient Items May Produce the So-Called Stimulus-Driven Capture

**DOI:** 10.3389/fpsyg.2018.00218

**Published:** 2018-02-23

**Authors:** Hanna Benoni

**Affiliations:** Department of Psychology, The College of Management Academic Studies, Rishon LeZion, Israel

**Keywords:** stimulus-driven, bottom-up attention, bottom-up salience, top-down attention, goal-driven, singleton, attentional capture, attentional control

## Abstract

The current study proposes that top-down attentional prioritization of salient items may produce the so-called stimulus-driven capture. To test this proposal, the “*expectation-based paradigm*” was designed on the basis of a visual search task. In Experiment 1, a task-irrelevant singleton frame was presented at the same location in 70% of the trials. The target was either presented at chance level within the singleton location, or away from it. In line with the singleton capture phenomenon, participants were faster in identifying the target when it appeared in the singleton location compared to non-singleton locations. However, leaving out the singleton frame in 30% of the trials led to a similar effect; participants were faster in identifying the target when it appeared in the expected singleton location compared to expected non-singletons locations (a “quasi-capture” effect). These results suggest that the participants allocated their attention to the expected singleton location, rather than that the singleton itself captured attention. In Experiment 2, the same task-irrelevant color singleton was presented in a random position in 70% of the trials. This color frame was shown as a non-singleton in all of the 30% singleton-absent multicolored trials. A similar facilitation effect was obtained when the target appeared in the expected singleton color frame compared to other frames, in singleton-absent trials as in singleton-present trials. These results further support the idea that instances of singleton capture can be explained by top-down attentional shifts toward singleton items. Theoretical implications of these results are discussed. Mostly, the study calls to consider the possibility that all sources of attentional control may be represented by a continuous variable of top-down control, including the category of “physical salience.”

## Introduction

A white swan in a bevy of brown ducks may receive your attention while you are sitting on the banks of a lake. According to models of attentional control, the swan is a salient item, and salient items that are different from their surroundings (also called “singletons”) tend to attract attention (e.g., Treisman and Gelade, 1980; Koch and Ullman, 1985; Julesz, 1986; Itti and Koch, 2000). The attentional shift toward the swan is labeled *bottom-up control of attention* in a *stimulus-driven manner* (also “exogenous” control), meaning that the stimulus itself provides the guidance, and the attentional deployment does not depend on the internal mental state of the observer, their knowledge, or their goals.

In a similar scenario, if you are looking for a white swan in a lake filled with various multicolored birds, you may find a swan by guiding your attention to the set of its characteristics (e.g., Wolfe et al., 1989; Wolfe, 1994). The attentional shift toward the swan is now labeled as *top-down control of attention* in a *goal-driven manner* (also “endogenous” control), meaning that your system provides the guidance according to your current goals, experience, and knowledge.

The world we view is composed of a vast amount of information that exceeds our processing capacity (e.g., Broadbent, 1958), and the brain must determine what information warrants the allocation of attention. The sources of attentional control determine which information will be prioritized and perceived and which will be ignored, and this ability is vital for many cognitive functions. For that reason, the dichotomy between bottom-up and top-down control of attention has been inherent to most theories of visual attention for decades (e.g., Jonides, 1981; Wolfe et al., 1989; Posner and Petersen, 1990; Wolfe, 1994; Kim and Cave, 1999; Itti and Koch, 2000).

### Stimulus-Driven Capture vs. Contingent Attention Capture

Some of the fundamental discussions in the study of attention have been spurred by the bottom-up vs. top-down dichotomy. For instance, over the past 25 years, a heated debate has emerged regarding the extent to which selection is controlled by top-down or by bottom-up factors, and especially how top-down and bottom-up mechanisms interact to set attentional priorities [see Burnham (2007), Theeuwes (2010), Theeuwes et al. (2010), Lamy et al. (2012) for reviews]. According to the *salience-based model* developed primarily by Theeuwes and colleagues (e.g., Theeuwes, 1992, 1994a; 1994b; Itti and Koch, 2001; Theeuwes and Godijn, 2002; Hickey et al., 2006), attention is first deployed to the most salient object within a spatially defined window of attention, mandatorily and irrespective of the observer’s goals. After this element has been selected, attention may move on to other locations based on task demands and top-down control settings.

This view has commonly relied on the *additional singleton task* developed by Theeuwes (1991, 1992). For example, participants searched for a green circle among green squares and had to report the orientation of the line inside the circle (form singleton). On half of the trials, one of the irrelevant squares was red (color singleton), whereas the others were green (Theeuwes, 1991). The results demonstrated that the presence of the irrelevant color singleton increased response time to the relevant shape singleton, indicating that irrelevant salient items involuntarily capture attention.

At the other end of the spectrum, *the contingent-capture account* (e.g., Folk et al., 1992; Folk and Remington, 1998; Folk and Anderson, 2010) emphasizes the role of top-down factors. According to this account, a salient stimulus will capture attention involuntarily only when the stimulus shares target-defining features, whereas it can be ignored when it does not match the attentional set of the observer.

This view has mainly relied on the *modified spatial cueing task* in which a cue display was followed in rapid succession by a target display. In the classic study by Folk et al. (1992), participants were required to identify a target singleton in a display of four possible locations. The search display consisted of either a color singleton (a red item among white ones) or an onset singleton (the only element in the display). Immediately preceding the target display by 150 ms, a cue display was presented. The cue display consisted of either a color cue (in which one location was surrounded by red dots and the other three locations were surrounded by white dots) or an onset cue (in which one location was surrounded by an abrupt onset of white dots and the remaining locations remained empty). The locations of the cue and target were uncorrelated; thus, the cue appeared at the same location as the target (“valid cue”) only in 25% of the trials (chance level). Attentional capture was measured as the performance benefit on trials where the target appeared at the same location as the cue vs. at a different location. The important finding was that task-irrelevant salient cues captured attention only when their unique property matched that of the singleton for which observers were searching.

In spite of intensive research and attempts to reconcile the aforementioned debate (e.g., Theeuwes et al., 2000; Becker, 2007; Belopolsky et al., 2007, 2010; Chen and Mordkoff, 2007; Sawaki and Luck, 2010; Carmel and Lamy, 2015), there is no consensus regarding the relative contributions of the physical salience of stimuli and of the observer’s goals in the allocation of attention.

### The Ambiguous Taxonomy of Attentional Control

Despite the broad dominance of the theoretical dichotomy between top-down and bottom-up attentional control, the boundaries of these two types are not always clear. Mostly, the presumed equivalence between “top-down” attentional control and the “goal-driven” manner yields a significant gap (Awh et al., 2012). Wolfe et al. (2003) proposed that top-down information can come in several other forms in addition to goal-driven; implicit information or knowledge can also be considered a form of top-down control. For instance, in the *priming of pop-out* paradigm studied by Maljkovic and Nakayama (1994, 1996), attention was more rapidly directed toward a red item if recent target items had also been red. Because this effect relies on what the observer has learned about prior trials (i.e., the observer’s implicit knowledge) and does not rely solely on the state of the stimulus, Wolfe and colleagues proposed that it can be considered a form of top-down guidance. In the same vein, Awh et al. (2012) emphasized how the tendency to equate top-down and goal-driven control over attention results in a taxonomy that cannot account for a wide range of phenomena that are unrelated to current selection goals and physical salience. For example, stimuli associated with high reward receive more attention than equally salient stimuli associated with low reward, even when this behavior contradicts current goals (Hickey et al., 2010). However, Awh and colleagues argued that since current goals and other implicit types of “top-down” control may generate conflicting selection biases, they should be viewed as distinct categories of control. The authors then proposed a *third* category of control termed “selection history.” Selection history refers to a lingering effect of past selection criteria that modulates the current attentional deployment (see also Bucker and Theeuwes, 2014; Stankevich and Geng, 2014; Munneke et al., 2015).

Thus, while discussing the gray area that defies the top-down vs. bottom-up basic dichotomy, some researchers (e.g., Wolfe et al., 2003) sort the sources of attentional control to bottom-up factors (i.e., “physical salience”) vs. a wide range of phenomena that can be considered as different types of top-down factors (implicit and explicit types), while others (Awh et al., 2012) classify the sources into three categories: bottom-up “physical salience,” “selection history,” and top-down “current goals.”

### The Criterion for Pure Stimulus-Driven Capture

In spite of the aforementioned debate and the ambiguity of the taxonomy of attentional control, no one questions the fact that the taxonomy of attentional control consists of a clear distinct category of bottom-up “physical salience.” Moreover, there is a broad agreement around the criterion to assess stimulus-driven capture. That is, there is an agreement that a hypothetical demonstration of attentional shifts toward a completely *task-irrelevant salient* item or event, that is also irrelevant in the sense that it does not share target-defining features, must reflect a bottom-up phenomenon governed by the properties of the stimulus display itself. As noted by Yantis and Egeth (1999), one can speak of selection in a *purely* stimulus-driven fashion when the stimulus feature in question is completely task-irrelevant, so that there is no incentive for the observer to attend to it deliberately. Furthermore, as expressed by Yantis and Egeth (1999), “If an object with such an attribute captures attention under these conditions, then and only then can that attribute be said to capture attention in a *purely stimulus-driven* fashion” (p. 663).

Note that although the contingent capture account (e.g., Folk et al., 1992) does not predict attentional shifts toward task-irrelevant salient stimuli that do not share target defining attributes, this account does not contend that such attentional shifts would reflect a bottom-up process in a stimulus-driven fashion either. Moreover, according to this approach “With a control setting established, an event exhibiting the critical properties will involuntarily summon attention, whether or not the event is actually relevant to task performance” (Folk et al., 1992, p. 1041). Thus, according to this account, the attentional shifts toward task-irrelevant singletons that do match target defining features are contingent on the observers’ attentional sets and are epiphenomenal to them, but essentially reflect a stimulus-driven process. As expressed by Burnham (2007) “By this account, control settings *mediate* capture, but this does not necessarily mean that they *cause* capture” (p. 410).

Therefore, it can be said that the notion of bottom-up control of attention in a stimulus-driven fashion is now part of the theoretical lexicon of every cognitive researcher in the area of visual attention and, accordingly, it is taken for granted that attention may be “captured by” or “driven by” physical properties that are external to the observer.

### The Current Study

The definition of bottom-up stimulus-driven capture relies on the assumption that the attentional system acts in full harmony with *task relevance*. Thus, salient stimuli that do not match the observer’s current goals are marked by the system as *completely irrelevant*. However, this assumption neglects the more reasonable possibility that singleton items, by being different, may be marked by the attentional system as *essentially relevant*. For instance, a salient singleton item is statistically more likely to be missed (one vs. many) than identical non-singleton items that provide redundant information. Therefore, to prevent information loss and to maximize information gain, it would be ecologically efficient if the attentional system will be tuned to prioritize the information that is prone to be missed (unique information). The idea that task-irrelevant salient information may be essentially relevant is also in line with previous assertions that salient items are more informative and that attention is predominantly guided to informative locations (e.g., Itti, 2007).

If unique items in the visual field are indeed *essentially relevant*, then attentional shifts toward all unique salient items (task-irrelevant as task-relevant) may reflect a type of top-down control of attention.

Thus, this study aims to question the undisputed definition of pure bottom-up control of attention in a stimulus-driven manner, and to expand the range of phenomena that can be considered top-down phenomena of attentional control. More specifically, the current study proposes that attention prioritizes and continuously seeks out unique items or events within the visual field in a top-down manner. In other words, attentional shifts toward task-irrelevant salient stimuli, in so-called stimulus-driven effects, may be initialized by a process that is *internal* to the observer and begins *before* the appearance of any external physical properties.

The current proposal shares similarities with both the salient-based account (e.g., Theeuwes, 1992) and the contingent capture account (e.g., Folk et al., 1992). With respect to the stimulus-driven account, the current proposal suggests that a salient stimulus has great potential to initially receive attentional resources even if it does not match the observer’s current goals and target-defining attributes. However, dissimilar to this account, the current proposal suggests that such effects may essentially reflect a top-down process. In this manner, the current proposal is consistent with the contingent capture account, which suggests that attention is largely dominated by top-down factors.

At the same time, the current proposal differs from both the stimulus-driven and the contingent capture accounts. The current study proposes a top-down factor that has not been proposed yet. It proposes that beyond current goals and similarities to target-defining features, attention continuously seeks out unique items and events in the visual field in a top-down manner. Hence, the current study questions possible effects that both the stimulus-driven and the contingent capture accounts would define as effects of stimulus-driven capture (i.e., demonstrations of attentional shifts to task-irrelevant salient items that do not share either target-defining attributes).

### Introducing the “Expectation-Based” Paradigm

To test the current proposal, the “*expectation-based” paradigm* was designed. The basic rationale was to design a condition in which task-irrelevant singleton is expected in a certain location or color, but is actually absent in part of the trials. And then, to test whether attention was directed to the location of this expected task-irrelevant singleton, irrespective of whether the singleton was actually presented. That is, to test whether attention was directed to the location of the expected singleton, even in singleton-absent trials, that cannot produce stimulus-driven capture.

In two variations of this paradigm, participants were instructed to search for a target (“K” or “H”) in a display of four letters that were presented within four frames, located up, down, left, and right in the visual field. Each experiment intermixed displays in which a singleton frame was presented (70%) or absent (30%). Participants received pre-knowledge of the singleton location (Experiment 1) or color (Experiment 2). In singleton trials, the target appeared in 25% of the trials within the singleton frame (chance level). Therefore, the pre-knowledge of the singleton-defining features was completely irrelevant to task demands and there was no incentive to deliberately start searching at the salient singleton. It should be emphasized that the singleton-defining feature was presented even in the non-singleton displays (e.g., if the singleton item in singleton trials was a white frame among red frames, then in non-singleton trials, a white frame was presented among multicolored frames). This design enabled the examination of top-down attentional shifts toward the singleton-defining feature in *non-*singleton trials.

According to salient-based accounts (e.g., Theeuwes, 1992), attentional shifts toward task-irrelevant singleton items are stimulus-driven; therefore, a “capture” effect (i.e., better performance in identifying a target that appears in a singleton location vs. other locations) is expected only in trials in which an irrelevant singleton is presented. In non-singleton trials, a singleton feature is absent; thus, capture effect is not expected to be obtained.

In the design of the current experiments, the target is not defined by specific features that only match the singleton item and not the other items in the display. Therefore, according to contingent-capture accounts (e.g., Folk et al., 1992), a “capture” effect is not expected in singleton-present trials. Albeit, demonstrations of such unexpected “capture” effects will be defined as stimulus-driven capture. In non-singleton trials, the display does not contain a unique stimulus. Therefore, all stimuli equally match or mismatch the target’s characteristics and capture effect is not expected to be obtained.

Alternatively, if the attentional shifts toward task-irrelevant singleton items are determined by a form of top-down attentional prioritization as proposed by this study, then it is expected that a “quasi-capture” effect will occur irrespective of whether the singleton is actually presented. That is, target facilitation will also occur when the target is presented in *expected* singleton locations compared to other locations, even in non-singleton trials.

Demonstrations of target facilitation when the target is presented in *expected* singleton locations compared to other locations, even in non-singleton trials, would reveal that attention is directed to *expected* singleton locations before the appearance of any given stimulus. If the processing of the stimulus is determined by events that *preceded* the stimulus, the processing could not have been driven by the stimulus.

## Experiment 1

**FIGURE 1:**
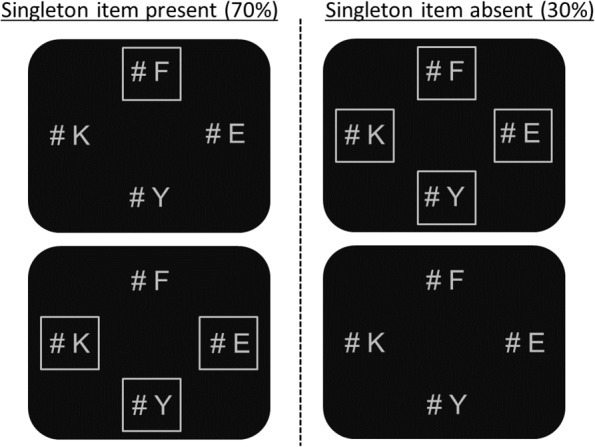
Examples of the stimulus displays used in Experiment 1. In these examples, the singleton item is presented in the expected upper location and the target (“K”) is presented in one of the expected non-singleton locations.

In the first experiment, the *location* of the irrelevant singleton was known in advance. Participants were instructed to search for a target (“K” or “H”) in a display of four letters. In singleton trials (70%), the singleton was defined by being the only letter surrounded by a frame or the only letter not surrounded by a frame. In non-singleton trials (30%), all letters were surrounded by frames or all letters were presented without frames (**Figure 1**). Participants completed four blocks. In each block, the singleton item was presented in the same location (up, down, left, or right) throughout the entire block. The target appeared in each location at chance level. Therefore, the pre-knowledge of the singleton location was completely irrelevant to task demands.

It should be noted that since not all paradigms demonstrate attentional shifts toward irrelevant singleton items (e.g., Folk et al., 1992; Jonides and Yantis, 1988), the design of this experiment (and the following experiment) followed Theeuwes and colleagues’ suggestion to maximize the sensitivity of the measurement. In each trial, all stimuli were presented simultaneously (e.g., Theeuwes et al., 2000, 2010). Furthermore, each presentation was characterized by a small set size and small viewing angle, conditions which are considered to be optimal for creating a diffused attentional window that encompasses the entire display at once (e.g., Theeuwes, 2004; Belopolsky et al., 2007; Theeuwes et al., 2010). In Experiment 1, the letters presented adjacent number-signs. The number-signs were included in the displays under the assumption that this addition would enhance the sensitivity of the measurement. So that it would be harder for attention to disengage from such stimuli and re-direct itself.

Finally, following Turatto et al. (2004) suggestion to increase the sensitivity of the measurements as well, the experiments use a simple search task. In a search task as such, attentional shifts toward an irrelevant singleton are normally inferred if reaction times (RTs) are faster in trials in which the target position coincides with that of the singleton, than when target and distractor singleton occupy different positions.

### Materials and Methods

#### Participants

The participants were 11 undergraduates (eight women and three men) aged 20–29 years from the College of Management – Academic Studies, Rishon LeZion, who participated to fulfill a course requirement. All had normal or corrected visual acuity and normal color vision, and were kept naïve with respect to the purpose of the experiment. The experiment was approved by the Institutional Review and Ethics Board of the college. The participants provided their written informed consent.

#### Apparatus

The stimuli were presented via Authorware software on a 17” monitor. Each participant was tested individually in a dimly lit room. Responses were collected via the computer keyboard. A chinrest was used to stabilize the viewing distance at 57.5 cm from the monitor so that 1 cm on the display represented 1° of visual angle.

#### Stimuli and Procedure

All stimuli were presented in a light gray color, RGB (170, 170, 170), on a black background. Participants searched for a target (“K” or “H”) in a display of four letters. The target subtended 0.55° of visual angle in height and 0.45° in width, and was presented randomly and with equal frequency in one of four possible locations centered at 2.6° to the right, to the left, above, or below the fixation. Three different neutral letters, identical in size to the target (0.55° × 0.45°), were randomly sampled from the set X, M, Y, A, F, and E, and placed in the three positions not occupied by the target. All letters presented adjacent number-signs subtended 0.55° in height and 0.45° in width. Each frame subtended 1.6° of visual angle from edge to edge. In singleton trials, one letter was the only stimulus surrounded by a frame or the only stimulus not surrounded by a frame. In non-singleton trials, either all letters were surrounded by frames or all letters were not surrounded by frames. The two types of singleton trials (70%) were randomly intermixed with the two types of non-singleton trials (30%) within each block.

Participants completed four blocks. In each block, the singleton item was presented in the same location (up, down, left, or right), throughout the entire block. Thus, the singleton’s location was known in advance.

Participants were instructed to respond as fast and as accurately as possible. They were directed to press the “L” key with their right index finger when the target was H, and the “A” key with their left index finger when the target was K. Participants also received pre-knowledge of the singleton location before each block. They were told that the target would appear in 25% of the trials within each location (chance level) and therefore the singleton frame is completely irrelevant to their task.

Each trial began with a 500-ms fixation cross (0.7° × 0.7°), followed by a 500-ms blank interval followed by the stimulus display that remained on the screen until a response. There was a 500-ms interval between trials. The four conditions were presented in a random order. Each block consisted of 200 trials grouped into four sub-blocks of 50 trials, and was preceded by 18 practice trials during which auditory feedback was given regarding accuracy.

### Results and Discussion

**FIGURE 2:**
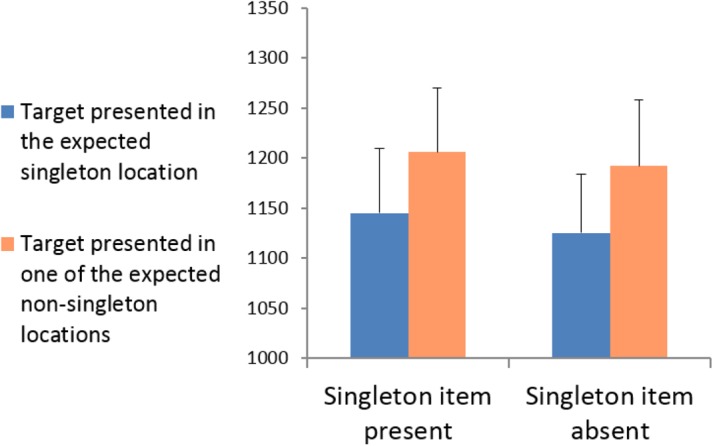
Experiment 1: Mean RTs for targets presented in the singleton location vs. targets presented in the non-singleton locations for singleton-present and singleton-absent displays.

Incorrect responses, and responses deviating by >2.5 standard deviations from the mean RT, calculated for individual participants and the relevant experimental condition, were removed from the RT analyses. A 2 × 2 repeated-measures analysis of variance (ANOVA) was conducted with singleton presentation (present vs. absent) and target location (presented in the expected singleton location vs. presented in one of the expected non-singleton locations). The mean RTs for each experimental condition are shown in **Figure 2**. The results revealed that the main effect of singleton presentation was not significant, *F*(1,10) = 1.73, *p* = 0.218, ${\text{η}}_{\text{p}}^{2}$ = 0.147; that is, RTs were equally fast in singleton-absent displays as in singleton-present displays. The main effect of target location was significant, *F*(1,23) = 33.974, *p* < 0.001, ${\text{η}}_{\text{p}}^{2}$ = 0.773: RTs were significantly faster when the target appeared in the *expected* singleton location (both when it was presented and absent) than when it appeared in the expected non-singleton locations. The interaction between singleton presentation and target location was not significant, *F*(1,10) = 0.024, *p* = 0.881, ${\text{η}}_{\text{p}}^{2}$ = 0.002, indicating that the same “capture” effect was obtained irrespective of whether the expected singleton was actually presented. That is, the same target facilitation was observed both when the target appeared at the actual singleton locations compared to non-singleton locations (“stimulus-driven capture”) and when all locations were similarly salient and the target appeared at a location that was only expected to be salient (expectation-based “quasi-capture”). These results support the idea that the obtained “stimulus-driven capture” arose solely from the expectation of a salient location rather than the physical saliency itself. That is, that the “stimulus-driven capture” is, in fact, a “quasi-capture.”

Although the lack of interaction between singleton presentation and target location indicates that the obtained target facilitation is similar in singleton and non-singleton trials, additional paired two-tailed *t*-tests were conducted to explore whether target facilitation is also significant in each condition separately. The analyses verify that both the “stimulus-driven capture” effect in singleton-present trials, *t*(10) = 2.280, *p* = 0.046, and the “quasi-capture” effect in singleton-absent trials, *t*(10) = 3.550, *p* = 0.005, were significant.

With respect to accuracy rates, the observers performed this task very well, with average performance at over 95% in each condition. An overall ANOVA performed on the accuracy data revealed that neither the main effect of singleton presentation, nor the main effect of target location, nor their interaction (all *p*s > 0.250) reached statistical significance. Thus, the RT findings do not appear to be compromised by a speed-accuracy trade-off.

#### Additional Analysis Including Singleton Type

In Experiment 1, there were two kinds of singletons; the location of the expected singleton could be framed (a framed location among non-framed locations; all framed locations) or non-framed (a non-framed location among framed locations; all non-framed locations). Thus, to demonstrate that the singleton status, and not a specific type of singleton, was responsible for the “capture” effect, the variable “singleton type” was included in the above ANOVA. The analysis conducted on mean RTs verifies that the singleton type did not interact with target location, *F*(1,10) = 0.039, *p* = 0.847, ${\text{η}}_{\text{p}}^{2}$ = 0.004; that is, the type of the singleton did not modulate the obtained “capture” effect. Additional analysis conducted on accuracy rates mimicked this finding, *F*(1,10) = 0.019, *p* = 0.893, ${\text{η}}_{\text{p}}^{2}$ = 0.002.

## Experiment 2

**FIGURE 3:**
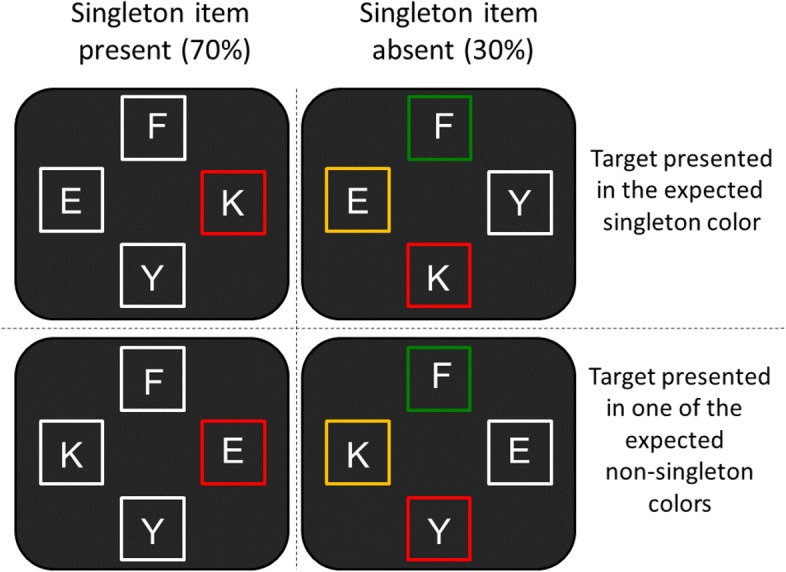
Examples of the stimulus displays used in Experiment 2. In these examples, the expected color singleton frame is red. Participants completed four blocks. In each block, one color was the singleton color throughout the block. Each color from the four options (red, yellow, green, and white) had its turn as the singleton color.

Experiment 2 followed the procedure of the first experiment except that *color expectation* was manipulated instead of location expectation. Participants were instructed to search for a target (“K” or “H”) in a display of four letters. In singleton trials, a singleton color frame was presented (e.g., a green frame among three yellow frames) in random positions. In non-singleton trials, the four frames appeared in four different colors (**Figure 3**).

### Materials and Methods

#### Participants

The participants were 16 undergraduates (12 women and 4 men) aged 22–28 years from the College of Management – Academic Studies, who participated to fulfill a course requirement or were paid 40 Israeli Shekels in exchange for their voluntary participation. All had normal or corrected visual acuity and normal color vision. The experiment was approved by the Institutional Review and Ethics Board of the college. The participants provided their written informed consent.

#### Apparatus

The apparatus was the same as that in Experiment 1.

#### Stimuli and Procedure

The possible colors for frames were white, RGB (255, 255, 255); green, RGB (0, 255, 0); yellow, RGB (255, 255, 0); and red, RGB (255, 0, 0). In all of the displays, the four letters were white and all of the letters were surrounded by frames. All letters subtended 0.75° of visual angle in height and 0.55° in width. Each frame subtended 1.3° of visual angle from edge to edge and centered at 2° from fixation. In non-singleton trials, the four frames appeared in the four different colors in random locations. In singleton trials, one of the frames was a color singleton, while the other three frames shared the same color. Participants completed four blocks. In each block, the singleton item was presented in a random location in the same color throughout the entire block (red singleton embedded among three white frames, white singleton embedded among three red frames, yellow singleton embedded among three green frames, or green singleton embedded among three yellow frames). Thus, the singleton’s color and the non-singleton colors were known in advance.

Participants were instructed to respond as fast and as accurately as possible. They were directed to press the “L” key with their right index finger when the target was H and the “A” key with their left index finger when the target was K. Participants also received pre-knowledge of the singleton color before each block. They were told that the target would appear in 25% of the trials within each location (chance level) and therefore the color singleton frame is completely irrelevant to their task.

Each block consisted of 200 trials grouped into four sub-blocks of 50 trials and was preceded by 18 practice trials during which auditory feedback was given regarding accuracy. In all other aspects, the stimuli and procedure were identical to those in Experiment 1.

### Results and Discussion

**FIGURE 4:**
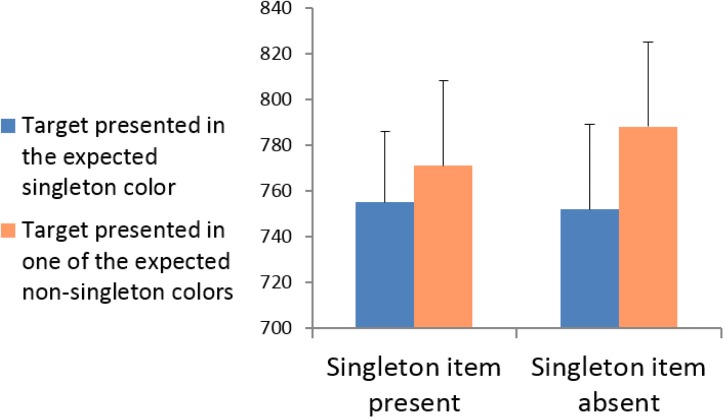
Experiment 2: Mean RTs for targets presented in the singleton color vs. targets presented in non-singleton colors for singleton-present and singleton-absent displays.

Incorrect responses, and responses deviating by more than 2.5 standard deviations from the mean RT, calculated for individual participants and the relevant experimental condition, were removed from the RT analyses. A 2 × 2 repeated-measures ANOVA was conducted with singleton presentation (present vs. absent) and target location (presented in the expected singleton location vs. presented in one of the expected non-singleton locations). The mean RTs for each experimental condition are shown in **Figure 4**. The results revealed that the main effect of singleton presentation was not significant, *F*(1,15) = 1.218, *p* = 0.287, ${\text{η}}_{\text{p}}^{2}$ = 0.075; that is, the target was identified equally as fast in singleton-absent displays as in singleton-present displays. The main effect of target location was significant, *F*(1,15) = 8.824, *p* = 0.010, ${\text{η}}_{\text{p}}^{2}$ = 0.370; RTs were significantly faster when the target appeared in the *expected* singleton location (both when it was presented and absent) than when it appeared in the expected non-singleton locations. The interaction between singleton presentation and target location was not significant, *F*(1,15) = 1.454, *p* = 0.247, ${\text{η}}_{\text{p}}^{2}$ = 0.088. Thus, attention was directed to expected singleton colors irrespective of whether the expected singleton was actually presented. In general, the results of Experiment 2 mimicked the results obtained in Experiment 1 and further support the idea that attention is largely governed by top-down factors, even in conditions presumed to be conditions of pure bottom-up control of attention.

Although the absence of an interaction between singleton presentation and target location indicates that the significant “quasi-capture” is statistically similar in singleton and non-singleton trials, additional paired two-tailed *t*-tests were conducted to explore whether target facilitation is also significant in each condition separately. Most importantly, the analyses verify that the “quasi-capture” effect in singleton-absent trials was significant, *t*(15) = 2.656, *p* = 0.018. However, the “stimulus-driven capture” in singleton-present trials did not reach significance, *t*(15) = 1.465, *p* = 0.163. These additional comparisons suggest that we may consider the non-singleton trials as a better condition for producing an observable “quasi-capture” effect. The significant “quasi-capture” effect that was obtained in non-singleton trials clearly indicates that attention was directed to expected singleton colors. Since participants have no pre-knowledge about the type of trial before it appears (whether it is a singleton or non-singleton trial), it is not plausible that attention was directed to expected singleton colors only in non-singleton trials. Thus, the differences between the simple effects must imply that the singleton trials were less sensitive to reveal the “quasi-capture” effect. A possible explanation to this pattern may be that in non-singleton trials which are constructed from four different multicolored heterogeneous frames, it is harder for attention to re-direct itself from the expected singleton locations to other locations compared to singleton trials, that consist of only two different colors. This explanation is in line with several claims that salient but irrelevant stimuli could draw attention without producing an observable capture effect if attention quickly disengaged from such a stimulus and re-directed itself (Theeuwes et al., 2000; Pratt and McAuliffe, 2002).

With respect to accuracy rates, the results indicated that only the main effect of singleton presentation was significant, *F*(1,15) = 5.932, *p* = 0.028, ${\text{η}}_{\text{p}}^{2}$ = 0.283; the accuracy rate was higher in trials with a singleton color frame (97.6%) than in multicolored trials without a singleton (97%). Neither the main effect of target location nor the interaction (all *p*s > 0.250) reached statistical significance. Thus, the accuracy results were in general agreement with the RT results.

#### Additional Analysis Including Singleton Color

Non-singleton trials in Experiment 2 were composed of four different color frames. Thus, even though in these displays there was not an odd color, one could still argue that the obtained “quasi-capture” effect may be accounted for one or two colors of higher luminance.

To answer this question, it should be emphasized that in Experiment 2, participants completed four blocks. In each block, one color (from the four color options) was the expected singleton color throughout the entire block. Each color from the four options (red, yellow, green, and white) had its turn as the singleton color. Thus, the expectation-based quasi-capture in non-singleton trials was tested by comparing the mean RTs of all the colors when they were expected to be singletons, to the mean RTs of all the colors when they were expected to be non-singletons. This comparison overrides the need to control luminance.

Notwithstanding, to rule out that one or two colors of higher luminance accounted for the obtained “capture” effect in both singleton-absent and singleton-present trials, the variable “singleton color” (red, green, yellow, and white) was included in the above ANOVA. The analysis conducted on mean RTs verifies that the color of the singleton did not interact with target location, *F*(3,45) = 0.578, *p* = 0.632, ${\text{η}}_{\text{p}}^{2}$ = 0.037; thus, the color of the singleton did not modulate the obtained “capture” effect. Additional analysis conducted on accuracy rates mimicked this finding, *F*(3,45) = 0.547, *p* = 0.653, ${\text{η}}_{\text{p}}^{2}$ = 0.035.

#### Additional Inter-trial Analyses

In Experiment 1, the singleton location was fixed and remained constant throughout each block. Therefore, it might be suggested that attention was drawn to the singleton location in non-singleton trials due to bottom-up location priming by the singleton item in the previous singleton trials. Similarly, in Experiment 2, the singleton color remained constant throughout each block. The fact that this pre-known color was a singleton color in singleton-present trials may enhance the perceptual sensitivity of this color. Therefore, it might be suggested that attention was drawn to this color in non-singleton trials due to bottom-up singleton color priming by the previous singleton trials.

Although such claims seem to be reasonable, a closer look at the paradigm and at the results makes this implausible for several reasons: (A) Most studies investigating priming [priming of pop out (PoP) and inter-trial feature priming (IFP)] reported better performance in visual search when the *target* feature was repeated across trials (e.g., Maljkovic and Nakayama, 1994). Moreover, Maljkovic and Nakayama (1994) found a facilitation effect when the target position was repeated in the consecutive trial and *inhibition* when a target was placed where a distractor used to be. In the current experiments, in non-singleton trials, when the target appeared at the singleton expected location (or in the singleton expected color), it was located in 75% of the cases where a distractor used to be and therefore was not expected to be facilitated in these trials compared to the other trials. (B) In Experiment 1, the singleton location was fixed, but the singleton-defining feature was not (the presentation of the frame). The stimuli in each location had switched in a similar fashion, from a letter that was not surrounded by a frame to a letter that was surrounded. Thus, the perceptual sensitivity is expected to be similar in each location in this experiment, and as a result, the exact carryover effect is expected in each location. (C) In Experiment 2, if attention in non-singleton trials had been drawn to the singleton color due to bottom-up singleton color priming by the previous singleton trials, a higher carryover effect would have occurred in singleton trials than in non-singleton trials. Thus, the “capture” effect would have been smaller in non-singleton trials compared to singleton trials. However, as reported above, not only was the “capture” effect statistically similar in singleton-absent trials as in singleton-present trials, but there was even a tendency for a stronger effect in non-singleton trials than in singleton trials, as reflected in the mean RTs. These results indicate that the “capture” effect resulted from the anticipation of a given color singleton but not from mere singleton color priming.

To further ensure that the expectation-based “quasi-capture” effect resulted from anticipations of a given color singleton or location, rather than from mere bottom-up priming, a cross-experiment inter-trial analysis was conducted only in non-singleton trials. The goal of this analysis was to examine whether the expectation-based “quasi-capture” that was obtained in non-singleton trials was modulated by the type of trials that preceded the non-singleton trials (singleton trials vs. non-singleton trials). A mixed three-way ANOVA was conducted with experiment as the between factor, the proceeding trial (singleton vs. non-singleton) as a within factor, and target location (presented at the expected singleton feature vs. presented at the non-singleton feature) as a within factor. The results revealed that the main effect of experiment was significant *F*(1,25) = 31.495, *p* < 0.001, ${\text{η}}_{\text{p}}^{2}$ = 0.557. Thus, the task in Experiment 1 was generally more difficult (1168 ms) than the task in Experiment 2 (786 ms). As expected, the main effect of target presentation was significant *F*(1,25) = 9.918, *p* = 0.004, ${\text{η}}_{\text{p}}^{2}$ = 0.284, revealing that target identification was easier when it presented in the expected singleton feature. Importantly, the interaction between target presentation and type of proceeding trial was not significant *F*(1,25) = 0.192, *p* = 0.665, ${\text{η}}_{\text{p}}^{2}$ = 0.008; indicating that a similar “capture” effect was obtained in trials that followed singleton trials (49) and in trials that followed non-singleton trials (38). All other interactions and effects did not reach statistical significance.

With respect to accuracy data, accuracy rates were equally high in all of the conditions (above 95% in each condition). The analyses reveal that neither the main effects nor the interactions reached statistical significance (all *p*s > 0.1). Thus, the accuracy results were in general agreement with the RT results.

Finally, in Experiment 1 the feature of the capturing position (frame or non-frame) could or could not be repeated from the preceding trial. Therefore, the effects in this experiment may be restricted to inter-trial repetition priming from preceding trials.

To rule out this possibility, a 2 × 2 × 2 repeated-measures ANOVA was conducted on mean RTs with the type of the singleton in the present trial (frame or non-frame location), the type of the singleton in the preceding trial (frame or non-frame location), and target location (presented at the expected singleton feature vs. presented at the non-singleton feature). The results revealed that the triple interaction was not significant *F*(1,10) = 0.275, *p* = 0.612, ${\text{η}}_{\text{p}}^{2}$ = 0.027; thus, the results verify that the obtained “capture” effect in this experiment was not modulated by inter-trial priming. Analysis conducted on accuracy rates mimicked this finding, *F*(1,10) = 1.119, *p* = 0.315, ${\text{η}}_{\text{p}}^{2}$ = 0.101. Taken together, these results seem to rule out bottom-up priming explanations.

## General Discussion

The present study was designed to test the hypothesis that attention is largely governed by top-down factors even in conditions presumed to be conditions of pure bottom-up control of attention by a stimulus-driven mechanism. More specifically, the current study proposes that since task-irrelevant salient items may be essentially relevant, the attentional system is tuned to prioritize and seek out continuously unique items or events within the visual field. Thus, attentional shifts toward task-irrelevant salient unique items may be initialized by a process that is *internal* to the observer and that begins *before* the appearance of any external physical properties.

To test this hypothesis, the “expectation-based paradigm” was designed. In two experiments, target facilitation was achieved when the target was presented in the *expected* singleton location (Experiment 1) or color (Experiment 2) compared to other locations, irrespective of whether the singleton was actually presented. Thus, a “quasi-capture” was obtained in singleton-absent trials that cannot produce stimulus-driven capture. Moreover, the magnitudes of the “*stimulus-driven capture*” in singleton-present trials and the “*quasi-capture*” in singleton-absent trials were similar. Hence, the obtained “stimulus-driven” capture arose solely from the expectation of a salient property rather than the physical saliency itself. Since the effects observed in this study depend on events that *preceded* the stimuli (expectations), the processing could not have been driven by the stimuli themselves or reflect factors that are external to the observer.

### Relevance to the Stimulus-Driven vs. Contingent Capture Debate

The proposal that stimulus-driven capture may be a type of top-down phenomenon shares similarities with both the stimulus-driven account (e.g., Theeuwes, 1992) and the contingent capture account (e.g., Folk et al., 1992). With respect to the stimulus-driven account, the current proposal suggests that a salient stimulus has great potential to receive attention even if it does not match the observer’s current goals and target-defining features. However, consistent with the contingent capture account, the current proposal suggests that attention is essentially dominated by top-down factors.

The current proposal may also suggest a general explanation to bridge the gap between stimulus-driven and contingent-capture accounts. Under specific circumstances, top-down current goals may contradict the permanent goal to perceive salient items (e.g., in a laboratory experimental session). When it is not possible to simultaneously fulfill all the goals, the outcome will be contingent upon the values that the system calculates for each goal under moment-to-moment changes and on the ability of attention to implement these priorities. Thus, current goals may be prioritized before the permanent goal to perceive salient items and vice versa. This hypothesis may explain why capture effect is not obtained in all the paradigms (e.g., Jonides and Yantis, 1988; Folk et al., 1992). Moreover, this hypothesis may suggest that an absence of the “capture” effect does not necessarily indicate that attention does not prioritize the salient item over other items in the visual field. This suggestion is in agreement with other accounts that emphasize the idea that some paradigms may not be optimal or sensitive enough to test attentional shifts toward salient items (e.g., Turatto et al., 2004).

### Open Questions and Limitations

The results of this study suggest that instances of singleton capture may reflect, in fact, a top-down phenomenon. Yet some questions may be raised concerning the specific kind of top-down influence or top-down mechanism that was obtained in this study. The conjecture of this study is that the attentional system is tuned to prioritize and continuously seek out unique items or events within the visual field, irrespective of whether they are task-relevant or not. Below I present some open questions that may still require clarification, and suggest alternative top-down explanations to the current proposal. Note that these open discussions do not contradict the basic general suggestion that top-down control of attention may produce the so-called stimulus-driven capture.

#### Singleton That Coincides with the Target at Chance Level

Although in the current experiments the singleton coincides with the target only at chance level, it is possible that participants may have believed that the singleton was predictive of the target. It has been claimed by several researchers that participants are not necessarily good at judging whether non-predictive events are indeed non-predictive (e.g., Becker, 2007), and thus choose to voluntarily attend to singletons, believing they are predictors. This caveat may be more substantial considering the fact that in this study the experiments always contained fixed elements of the singleton (i.e., location or color). Thus, these fixed elements may encourage the observers to deliberately take the irrelevant singleton as a convenient starting point in their search (e.g., Todd and Kramer, 1994; Becker, 2007).

Against such claims it should be noted that the participants were informed explicitly that the target will appear at the singleton location only at chance level, and therefore, the singleton frame is completely irrelevant to their task. Moreover, such non-contributing strategy is not expected in small display sizes (e.g., Todd and Kramer, 1994) which are optimal for creating a diffused attentional window that encompasses the entire display at once (e.g., Theeuwes, 2004; Belopolsky et al., 2007; Theeuwes et al., 2010). Still, to rule out this possibility, additional subsequent experiments should be designed. For example, experiments that manipulate the probability of the target appearing at the singleton location, particularly, manipulations that reduce the probability that target and irrelevant singleton share a position below chance level.

#### The Expectation-Base “Quasi-Capture” vs. “Surprise-Capture”

It has been demonstrated that a color singleton can capture attention in the complete absence of any anticipation to the singleton, that is, when the singleton is presented for the first time without prior announcement and following a number of trials without a singleton. This phenomenon is the so-called “surprise-capture” (e.g., Horstmann, 2002, 2005).

At first glance, the “surprise-capture” effect seems to contradict the expectation-based “quasi-capture” effects which obtained in this study, as the surprise capture suggests that events that deviate from expectations attract attention. However, these two effects should not be seen as being in conflict. The current study attempts to suggest that the attentional system is tuned permanently to seek unique items and events. The expectations that were provided in the current experiments enable to test top-down attentional shifts to salient items, but do not suggests necessarily that only expected singletons receive attention. On the contrary, the results obtained in this study suggest that each salient item or event has a great potential to receive attention.

Moreover, in a laboratory experimental session in which the properties of the irrelevant singleton are known in advance (as in the current “expectation-based paradigm”), top-down current goals may compete with the tendency of attention to seek unique salient items. In cases like this, when it is not possible to simultaneously fulfill all the goals, the outcome must depend on the values that the attentional system calculates for each goal. Therefore, current goals may receive higher prioritization compared to the permanent goals to perceive salient items. Accordingly, the “quasi-capture” effect is expected to be smaller or harder to achieve, compared to “surprised-capture” effect.

#### The Role of Expectation in the Obtained “Quasi-Capture” Effects

The hypothesis of this study was that attention prioritizes unique salient items in the visual field in a top-down fashion. Thus, even effects that presumed to be bottom-up effects of attention may be, in fact, effects of top-down control of attention. To test this hypothesis, the “*expectation*-based paradigm” was designed. Results have shown that attention was directed to expected singleton properties *before* the appearance of any given stimulus, these results cannot have been driven by the stimuli themselves. Hence, this study proposes that attention is essentially governed by top-down factors, even in conditions presumed to be conditions of pure bottom-up control of attention in a stimulus-driven manner.

However, it remains unclear whether the effects that were obtained in this study are restricted to conditions in which observers anticipate specific irrelevant singletons, or whether the attentional system is tuned to seek out permanently unique items in the visual field, even without anticipation of specific characteristics of singletons.

The direct conclusion that derives from the obtained results is that whenever the observer anticipates a certain singleton, the attentional system will prioritize this singleton and will be directed to its characteristics, whether or not this singleton is task-relevant. Moreover, that instances of singleton capture can be explained by expectations, rather than that the singletons themselves captured attention.

However, this study attempts to suggest a more radical proposal. The results obtained in this study might imply that the attentional system is permanently seeking out unique and salient items in the visual field, in a top-down manner, even without specific anticipations. Further research which manipulates anticipations should be conducted, to examine the role of expectations in the obtained “quasi-capture” effects.

### Concluding Remarks

Lastly, this study proposes that the human attentional system acts fully in accordance with different types of relevance, beyond current goals, and largely succeeds in prioritizing important information. Relevant information can come in several forms other than explicit current goals. For instance, information may be relevant based on the history of prior selections (e.g., Hickey et al., 2010; Awh et al., 2012; Yashar et al., 2017), permanent self-related goals such as one’s own name (e.g., Moray, 1959; Bargh, 1982), culture-related goals (e.g., Brosch and Sharma, 2005), or even phylogenetically implicit goals to perceive ecologically important stimuli such as potentially dangerous stimuli (e.g., Öhman et al., 2001; Flykt, 2005; New et al., 2007; Penkunas and Coss, 2013; Yorzinski et al., 2014) or salient, unique stimuli, as this study suggests.

All of these forms, as forms of relevance, reflect factors that are internal to the observer. By also considering “physical salience” as a form of relevance that is internal to the observer, this study proposes that attention deployment may be executed solely by different types of top-down priorities.

A lot of research needs to be done to further investigate this hypothesis and many questions remain unanswered. Yet, as a preliminary study, this work seeks, first and foremost, to raise new questions; it invites re-examination of the current dogma that the category of bottom-up physical salience is essentially separated from top-down factors of attentional control. Likewise, it invites a deliberation of any use of the canonical expressions that define attention as being “captured by” or “driven by.”

Instead, it calls to consider the possibility that all the factors of attentional control may be represented by a continuous variable of top-down control, including the category of “physical salience” that may signify the edge of the spectrum of this variable.

## Ethics Statement

This study was carried out in accordance with Helsinki guidelines. All subjects gave written informed consent in accordance with the Declaration of Helsinki. The protocol was approved by the Institutional Review Board of the College of Management –Academic Studies.

## Author Contributions

HB conceived the idea for the study, designed the experiments, and wrote the manuscript. HB also programed the experiments, collected most of the data, and performed the data analysis.

## Conflict of Interest Statement

The author declares that the research was conducted in the absence of any commercial or financial relationships that could be construed as a potential conflict of interest.
